# Trends in Alcohol Use Before and During the COVID-19 Pandemic Among Women Living With and Without HIV in the United States (2017–2022)

**DOI:** 10.1007/s10461-025-04875-9

**Published:** 2025-10-10

**Authors:** Hannah R. Tierney, Yifei Ma, Fan Xia, Aruna Chandran, Mirjam-Colette Kempf, Lauren F. Collins, Jack DeHovitz, Ralph J. DiClemente, Michelle Floris-Moore, Audrey L. French, Deborah L. Jones, Anjali Sharma, Amanda B. Spence, Judith A. Hahn, Jennifer C. Price, Phyllis C. Tien

**Affiliations:** 1 Department of Medicine, University of California, San Francisco, 505 Parnassus Ave # M1480, San Francisco, CA 94143-0454 USA; 2 Department of Medicine, University of California San Francisco, San Francisco, CA USA; 3 Department of Epidemiology and Biostatistics, University of California, San Francisco, San Francisco, CA USA; 4 Department of Epidemiology, Johns Hopkins University, Bloomberg School of Public Health, Baltimore, MD USA; 5 Schools of Nursing, Medicine and Public Health Birmingham, University of Alabama at Birmingham, Alabama, USA; 6 Division of Infectious Diseases, Emory University School of Medicine, Atlanta, GA USA; 7 Department of Medicine, State University of New York-Downstate Medical Center, Brooklyn, NY USA; 8 Department of Social and Behavioral Sciences, NYU School of Global Public Health, New York, NY USA; 9 Division of Infectious Diseases, University of North Carolina School of Medicine, Chapel Hill, NC USA; 10 Department of Medicine, Division of Infectious Diseases, Stroger Hospital of Cook County, Chicago, IL USA; 11 Department of Psychiatry and Behavioral Sciences, University of Miami Miller School of Medicine, Miami, FL USA; 12 Department of Medicine, Albert Einstein College of Medicine, New York, NY USA; 13 Division of Infectious Diseases, Georgetown University Medical Center, District of Columbia, Washington, USA; 14 Division of HIV, Infectious Diseases, and Global Medicine, University of California San Francisco, San Francisco, CA USA; 15 Department of Medicine, Department of Veteran Affairs Medical Center, University of California San Francisco, San Francisco, CA USA

**Keywords:** COVID-19 pandemic, HIV, Alcohol use, Alcohol consumption, Women

## Abstract

Data on alcohol use trends in women living with HIV (WLWH) are lacking. We examine data collected before and across the COVID-19 pandemic (2014–2022) in WLWH and socio-demographically similar women living without HIV (WLWOH)(*n* = 1564; 67% WLWH) who self-reported alcohol use to establish and compare trends. Women were categorized into baseline groups based on use pattern from 10/2014 to 09/2017: abstinence (no alcohol at any visit), non-heavy drinking (0 heavy drinking visits), some heavy drinking (1–4 visits), and persistent heavy drinking (5–6 visits) with heavy drinking defined as > 7 drinks/week or > 3 drinks/day. A linear mixed model (LMM) was used to assess for changes in average number of drinks/week (ADW) by time segment: pre-pandemic (10/2017-09/2019), early pandemic (08/2020-05/2021), and late pandemic (06/2021-09/2022). Time, baseline group, and HIV status were included as interactions. In the LMM, WLWH with persistent heavy drinking histories had lower ADW compared to WLWOH. There were no significant differences in ADW in the other groups by HIV status and no significant interactions between HIV status and the time segments. The largest change in ADW occurred among the persistent heavy drinking group, with decreases at pandemic onset followed by increases into 2021. Overall, WLWH reported less alcohol use than WLWOH, but changes in alcohol use trends from 10/2017 to 09/2022 were similar regardless of HIV status. This suggests that the pandemic did not uniquely impact alcohol use among WLWH, rather socio-economically disadvantaged women generally. These findings support the need for greater research in the role of socioeconomic disruption on alcohol use among women.

**Supplementary Information:**

The online version contains supplementary material available at 10.1007/s10461-025-04875-9.

## Background

Unhealthy alcohol use is a leading cause of preventable death among adults in the United States (U.S) [1]. Although alcohol use is less common in women than men, alcohol use, alcohol-related healthcare costs, and alcohol-related deaths have been increasing among women in the past several decades [2–4]. Women living with HIV (WLWH) have higher rates of alcohol use than their seronegative peers, with 13–21% of WLWH reporting heavy alcohol use compared to 9% among women in the general U.S. population [5–7]. Among WLWH, alcohol use is associated with decreased adherence to HIV antiretroviral therapy and unsuppressed HIV [8–10] as well as increases in HIV transmission risk behaviors [11–13]. Therefore, prevention and treatment of unhealthy alcohol use in this population is an important strategy for preventing new HIV infections and optimizing care across the HIV care continuum [14]. Yet, little is known about the impact of the coronavirus disease 2019 (COVID-19) pandemic on alcohol use trends among U.S. WLWH and how these trends compare to women living without HIV (WLWOH).

In the U.S., shortly after many communities issued stay-at-home orders during the early phase of the COVID-19 pandemic, national alcohol sales increased by 34% [15]. Cross sectional studies and “before” and “after” survey data from national samples of adults showed increases in self-reported alcohol use that were greatest among women [16–18]. These data contrast with a study of men and women living with HIV that found a decrease in binge drinking episodes early in the COVID-19 pandemic [19].

A limitation of these studies is that changes in alcohol use were measured over a short period of time and were not contextualized using longer-term pre-pandemic alcohol trends. Therefore, it remains unclear if changes in alcohol use early in the pandemic were a pandemic effect or part of a longer-term trend. Additionally, prior studies are limited to early in the pandemic; use trends following the wide-spread availability of SARS-CoV-2 vaccines in the U.S. are lacking, especially for WLWH. Participants also were not stratified based on their alcohol consumption history, which is relevant because prior alcohol use is predictive of future alcohol use [20, 21]. As a result of aggregate analysis approaches that don’t account for baseline differences in alcohol use, there could be masking of different alcohol use patterns that exist among sub-populations.

This study addresses several of these gaps by utilizing longitudinal data from the multisite Women’s Interagency HIV Study (WIHS). Our primary aim was to describe trends in self-reported alcohol use from 2017 to 2022 among WLWH and demographically similar women living without HIV (WLWOH) while accounting for earlier alcohol use history. We also hypothesized that changes in alcohol consumption following the COVID-19 pandemic would vary based on alcohol use history and HIV status.

## Methods

### Data Source

WIHS is the largest and longest running HIV cohort study of women in the U.S. It is demographically representative of WLWH nationally. Socio-demographically similar WLWOH were also enrolled. Recruitment was in a 2 WLWH to 1 WLWOH fashion. In 2019, the WIHS and the Multicenter AIDS Cohort Study (MACS) of men living with and without HIV, merged to create the MACS/WIHS Combined Cohort Study (MWCCS). There are 13 MWCCS sites across the U.S., 9 of which were originally WIHS sites. Additional details about the WIHS, recruitment of participants, and study instruments and procedures are described elsewhere [22, 23]. Data collection forms are publicly available (https://statepi.jhsph.edu/mwccs/). Clinical assessments and self-report surveys were completed on a semi-annual basis prior to the merger and now are conducted on an annual basis. From April to September 2020, participants were contacted to complete questionnaires by phone due to the need for remote contact in response to the COVID-19 pandemic. In-person annual visits resumed October 2020. Visits, including the phone questionnaires, were conducted in English and Spanish and participants were compensated.

### Compliance with Ethical Standards

The WIHS and now MWCCS is approved by an Institutional Review Board at each study site. Written consent is obtained from all participants.

### Study Sample

**Fig. 1:**
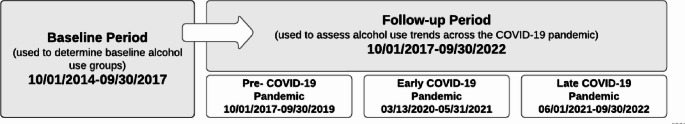
Analysis time periods. There was a total of 1564 women included in the study

This analysis includes data collected from October 2014 to September 2022. We defined the period October 2014–September 2017 as the baseline period and October 2017–September 2022 as the follow-up period. The baseline period began in October 2014, because new WIHS sites in the Southern U.S. were added in 2013 with enrollment of most participants by 2014. Only participants with available baseline period alcohol data were included in the study. Participants who had baseline data, but no follow-up data were excluded. Participants who persistently did not drink alcohol across both study periods were excluded from the analysis since we were most interested in changes in alcohol consumption over time. These time frames are illustrated in Fig. 1 and study timepoints are outlined in Supplemental Table 1.

## Measures

### Alcohol Consumption

Our primary measure is the number of alcoholic drinks per week. The frequency and quantity of alcohol use was assessed at each study visit. Participants were asked to recall how often they had a drink containing alcohol since their last study visit. During the first survey after the start of the pandemic (i.e., March to September 2020) the question asked about alcohol use since the pandemic start. Responses were ordinal (e.g. “at least once a day”, “nearly every day”, “3–4 times a week”, “1–2 days a week”, “1–2 times a month”, “about once a month”, “6–11 times a year”, “1–5 times a year”, “never”). If participants reported any alcohol use, they were asked how many drinks they had containing alcohol on a typical day when drinking. Responses were numeric (e.g. “1”, “2”, “3”, “4”, “5”, “6”, “7–9” and “10 or more”). Responses were used to calculate the average number of drinks per week since the last visit or pandemic start. Due to the structure of the data collection the maximum number of drinks per week captured was 77.

### Demographic and Clinical Measures

Demographic measures included age, study site, race and ethnicity, employment, annual household income, and marital status. Clinical variables included HIV status and number of chronic medical comorbidities. These measures were selected based on prior associations with alcohol use. Medical comorbidities were ascertained using definitions adopted from previously published WIHS data (Supplemental Table 2) [24]. Each participant was assessed for eight common comorbidities (not including HIV status) and the number of conditions present was totaled to represent the overall comorbidity burden. Given that demographic data is often stable over time and the medical comorbidities are chronic in nature, data were carried forward from a prior visit if they were missing.

### Statistical Analysis

#### Baseline Alcohol Use

Using data collected over 6 semiannual study visits during the baseline period (October 2014–September 2017), we created groups based on historic alcohol use patterns. We used this approach because participants with different historical alcohol use patterns likely have different ongoing alcohol use trends. To establish baseline alcohol use groups, we calculated the number of semiannual visits at which a participant met criteria for heavy drinking utilizing the National Institute on Alcohol Abuse and Alcoholism definition of > 7 drinks per week or > 3 drinks per day for women [25]. Baseline groups included abstinence (no alcohol at any of the 6 visits), non-heavy drinking (no visits with > 7 drinks per week or > 3 drinks per day), some heavy drinking (1–4 visits with > 7 drinks per week or > 3 drinks per day), and persistent heavy drinking (5–6 visits with > 7 drinks per week or > 3 drinks per day) (Table 1.). Descriptive statistics, Kruskal-Wallis tests, and chi-squared tests were used to compare demographic and clinical differences between women living with and without HIV and across baseline alcohol use groups.

**Table 1:** Baseline alcohol use group definitions

Group	Criteria
Abstinence	No alcohol consumption across any baseline visits
Non-heavy drinking	Some visits with 7 or less drinks per week and 3 or less drinks on one occasion; no heavy drinking visits
Some heavy drinking	At least one visit with more than 7 drinks per week or more than 3 drinks on one occasion
Persistent heavy drinking	At least 5 visits (out of 6) with more than 7 drinks per week or more than 3 drinks on one occasion

#### Alcohol Trends Before and During the COVID-19 Pandemic

Follow up period data (October 2017–September 2022) were examined using linear mixed models (LMM) to assess for changes in alcohol use (i.e. average number of drinks per week) immediately before (pre) and during the COVID-19 pandemic. LMM provide comparisons between trends pre- and during-pandemic while accounting for correlation within participants. They also provide unbiased estimates when data are missing at random. The fixed effect model included an interaction between baseline alcohol use groups and time. The correlation between repeated measures for participants over time was accounted for by including a random intercept and random slope for individuals in the random effect model. To assess for statistical differences in alcohol trends between WLWH and WLWOH, we examined one LMM that included an additional interaction for HIV status. We also stratified our analysis based on HIV serostatus and ran individual models for WLWH and WLWOH for ease of interpretation. In addition to examining trends, we developed a LMM adjusting for age, race and ethnicity, employment, annual household income, education, marital status, tobacco use, and the medical comorbidity burden. We calculated the within-study site and within-subject intraclass correlation coefficients (ICC) to measure the homogeneity within the sites and participants using linear mixed effects models. The intraclass correlation analysis based on the LMM showed that the within-site ICC was negligible (1.8%) and within-subject ICC was substantial (58.4%). Therefore, we decided not to include site in our models.

Three time segments were assessed through piecewise regression: pre-COVID-19 pandemic (October 2017–mid-March 2020), early pandemic (mid-March 2020–May 2021), and late pandemic (June 2021–September 2022). March 13th, 2020 (the start of the public health emergency in the U.S.) was used as the beginning of the early pandemic time segment. May 31st, 2021 was used as the end of the early pandemic segment because at this time vaccines were widely available and most states had completed all stages of reopening, both of which could influence social, economic, and drinking behaviors [26]. Knots were used to allow for flexibility between time segments. Trends were assessed for changes with potential clinical or public health significance. Statistical significance based on p-values was not emphasized.

#### Missing Data

During the baseline period (October 2014–September 2017), estimated values for missing alcohol use data were generated using multiple imputation by chained equations (MICE) from the available clinical and demographic and alcohol use data [27]. During the follow-up period (October 2017–September 2022), we assessed the correlation between percentage of missing alcohol use data and the mean average number of drinks per week for each participant. There was no correlation between a participant’s proportion of visits with missing alcohol data and their mean number of drinks per week (correlation coefficient − 0.01) so imputation was not performed for the follow-up period data because the missing at random assumption was met. SAS Version 9.4 and the MICE R package were used for the analysis.

## Results

Of the 2485 participants in the sample, we excluded 231 with no follow-up period data and an additional 690 participants (30.6%) who reported no alcohol use at all visits during the follow-up period, resulting in 1564 participants and 9578 included observations (Supplemental Fig. 1). There were missing alcohol use data in 6% of the included observations.

Table 2 shows the demographic and clinical characteristics of included participants (i.e. those reporting any alcohol use over the study time period). The majority were living with HIV (66.9%) and self-identified as being of Black/African American race (64.0%), having a single marital status (69.9%), and being unemployed (57.0%). Nearly half reported an annual household income of <$12K/year (46.0%). During the baseline period participants were categorized into the following drinking groups: abstinence (9.3%), non-heavy drinking (63.1%), some heavy drinking (18.3%), and persistent heavy drinking (9.3%). In analyses by HIV status, WLWH were more likely to be in the abstinent group (11.2% vs. 5.4%) and less likely to be in the persistent heavy drinking group (7.8% vs. 12.4%) compared to WLWOH (*p* < 0.001). Analyses of participant characteristics by baseline alcohol use group are shown in Supplemental Table 3.

**Table 2:** Demographics and clinical characteristics of women living with and without HIV in the Women’s Interagency HIV study

	Total	Women Living with HIV	Women Living without HIV	*P*-value
Total number	1564	1047	517	
**N (%) or mean ± sd**				
Baseline Alcohol Use Groups				
Abstinence	145 (9.3%)	117 (11.2%)	28 (5.4%)	< 0.001
Non-heavy consumption	987 (63.1%)	681 (65.0%)	306 (59.2%)	
Some heavy consumption	286 (18.3%)	167 (16.0%)	119 (23.0%)	
Persistent heavy consumption	146 (9.3%)	82 (7.8%)	64 (12.4%)	
Age, years	49.4 ± 9.33	50 ± 9.19	48.3 ± 9.5	< 0.001
Study Site				
Bronx	205 (13.1%)	117 (11.2%)	88 (17.0%)	< 0.001
Brooklyn	225 (14.4%)	155 (14.8%)	70 (13.5%)	
Washington DC	190 (12.1%)	120 (11.5%)	70 (13.5%)	
San Francisco	206 (13.2%)	131 (12.5%)	75 (14.5%)	
Chicago	179 (11.4%)	123 (11.7%)	56 (10.8%)	
Chapel Hill	129 (8.2%)	91 (8.7%)	38 (7.4%)	
Atlanta	196 (12.5%)	133 (12.7%)	63 (12.2%)	
Miami	75 (4.8%)	53 (5.1%)	22 (4.3%)	
Birmingham	76 (4.9%)	59 (5.6%)	17 (3.3%)	
Jackson	83 (5.3%)	65 (6.2%)	18 (3.5%)	
Marital Status				
Single	1048 (69.9%)	717 (71.5%)	331 (66.6%)	0.052
Married/Partnered	452 (30.1%)	286 (28.5%)	166 (33.4%)	
*Missing*	*64*	*44*	*20*	
Race				
Black/African American	1001 (64.0%)	680 (64.9%)	321 (62.1%)	0.085
White	163 (10.4%)	116 (11.1%)	47 (9.1%)	
Other	400 (25.6%)	251 (24.0%)	149 (28.8%)	
Hispanic Ethnicity	207 (13.2%)	126 (12.0%)	81 (15.7%)	0.046
Employed	669 (43.0%)	425 (40.7%)	244 (47.7%)	< 0.001
*Missing*	*7*	*2*	*5*	
Annual Household Income				
<$12,000	665 (46.0%)	453 (46.6%)	212 (44.9%)	0.260
$12,000–30,000	423 (29.3%)	292 (30.0%)	131 (27.8%)	
>$30,001	357 (24.7%)	228 (23.4%)	129 (27.3%)	
*Missing*	*119*	*74*	*45*	
Educational Attainment				
No high school diploma	470 (31.6%)	318 (31.5%)	152 (31.7%)	0.950
High school diploma	445 (29.9%)	304 (30.1%)	141 (29.4%)	
Some college or more	574 (38.5%)	387 (38.4%)	187 (39.0%)	
*Missing*	*75*	*38*	*37*	
Medical comorbidity burden	3.20 ± 1.96	3.37 ± 1.96	2.86 ± 1.91	<0.0001
Tobacco smoking				
Never	471 (31.5%)	341 (33.8%)	130 (26.9%)	0.019
Former	405 (27.1%)	271 (26.8%)	134 (27.7%)	
Current	618 (41.4%)	398 (39.4%)	220 (45.5%)	
Visits completed during the follow-up/COVID-19 time period	5.76 ± 1.52	5.82 ± 1.45	5.63 ± 1.66	0.120

The Kruskal-Wallis test was used to compare means across baseline alcohol use groups. Chi-squared tests were used to compare proportions across baseline alcohol use groups

In the LMM of all women included in this analysis with HIV as an interaction term, WLWH with a history of persistent heavy drinking consumed less alcohol on average compared to WLWOH from October 2017 to September 2022 (-8.75 average drinks per week, 95% CI -13.31, -4.20, *p* < 0.001) (Supplemental Table 4). Despite differences in the amount of alcohol use, changes in the average drinks per week over time did not statistically differ by HIV status.

**Fig. 2:**
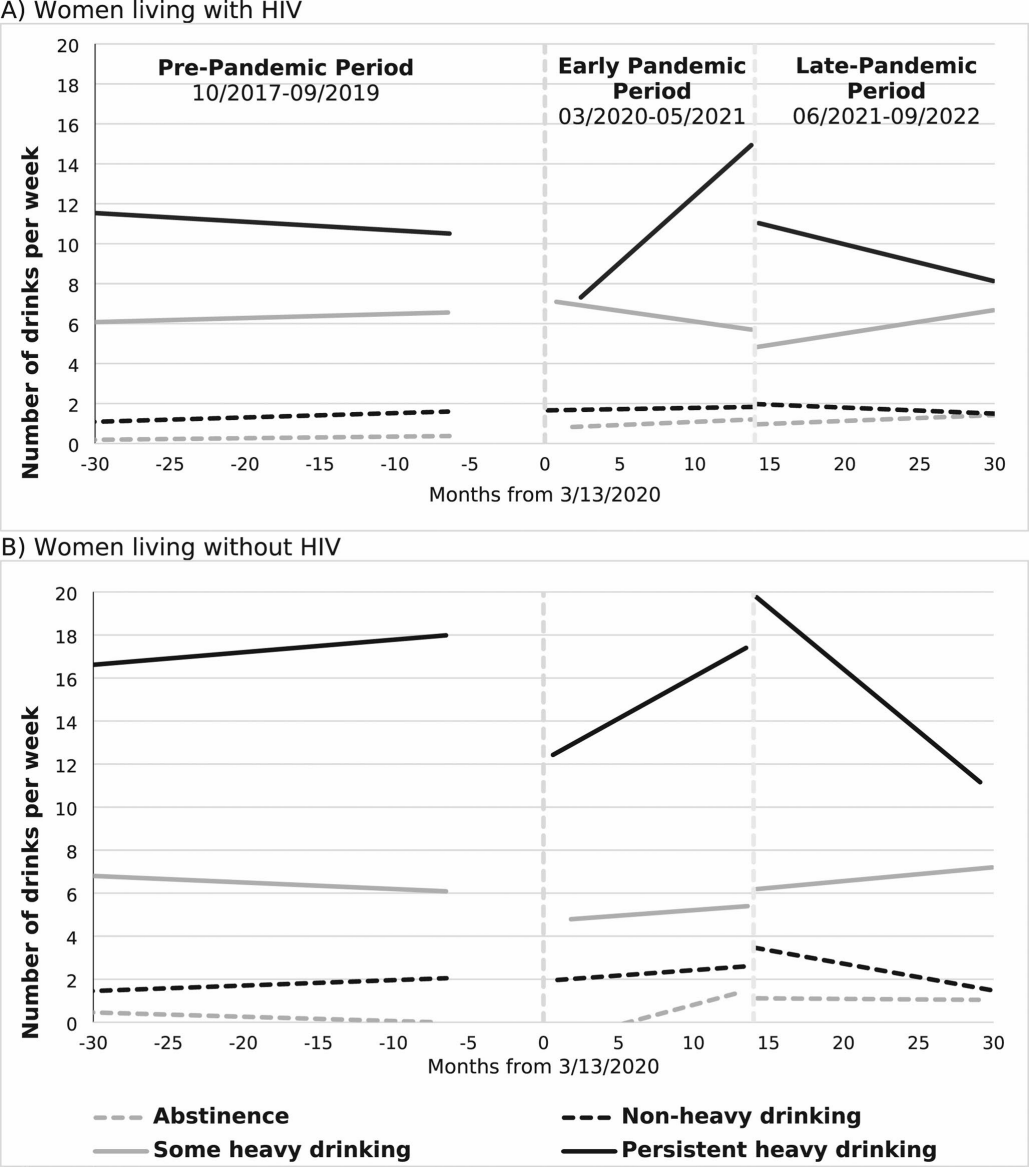
Linear mixed model fitted values illustrating trends in number of drinks per week before and during the COVID-19 Pandemic (2017–2022) among women living with and without HIV, by baseline (2014–2017) alcohol use group

LMM trends stratified by HIV status are shown in Fig. 2; Table 3. The model outputs used to generate the trends are in Table 4. For WLWH and WLWOH, the mean number of drinks per week during the pre-pandemic period were mostly stable across all baseline drinking groups. Among those with a history of abstinence, there were increases in the average number of drinks per week observed across the early pandemic period that persisted in the late pandemic period. Among those with a history of non-heavy drinking, average drinks per week remained mostly stable across all time periods. For women with a history of some heavy drinking, the average number of drinks per week were stable across the pre-pandemic and early pandemic time periods, but there was a slight increase in alcohol use across the late pandemic period. For women with a history of heavy drinking there was a decrease in the average number of drinks per week at the early pandemic onset compared to pre-pandemic levels of alcohol use. However, alcohol use increased throughout the early pandemic period resulting in an increase in the average number of drinks per week to at or above pre-pandemic levels. This change did not persist into the late pandemic period where the average alcohol use in both groups decreased below pre-pandemic levels.

**Table 3:** Linear mixed model estimates of the number of drinks per week before and during the COVID-19 pandemic (2017–2022) among women living with and without HIV

Baseline Group			Pre-pandemic	Early Pandemic	Late pandemic
Abstinence	HIV+	Average at period start, mean (SD)*	0.18 (1.61)	0.83 (2.10)	0.96 (1.83)
		Slope during the period (95% CI)**	0.008 (−0.053, 0.069)	0.031 (−0.194, 0.257)	0.029 (−0.193, 0.251)
	HIV−	Average, mean (SD)	0.47 (2.21)	0.00 (2.27)	1.11 (3.15)
		Slope (95% CI)	−0.020 (−0.174, 0.134)	0.187 (−0.416, 0.791)	−0.005 (−0.576, 0.566)
		Slope Difference (95% CI)***	0.027 (−0.122, 0.177)	−0.156 (−0.735, 0.423)	0.034 (−0.517, 0.585)
Non-heavy drinking	HIV+	Average, mean (SD)	1.07 (1.57)	1.66 (1.83)	1.97 (1.61)
		Slope (95% CI)	0.022 (−0.068, 0.112)	0.013 (−0.322, 0.347)	−0.300 (−0.355, 0.295)
	HIV−	Average, mean (SD)	1.44 (2.03)	1.97 (2.44)	3.47 (2.14)
		Slope (95% CI)	0.025 (−0.198, 0.249)	0.050 (−0.823, 0.924)	−0.125 (−0.949, 0.699)
		Slope Difference (95% CI)	−0.031 (−0.189, 0.127)	−0.135 (−0.684, 0.414)	0.062 (−0.516, 0.639)
Some heavy drinking	HIV+	Average, mean (SD)	6.06 (1.81)	7.09 (1.86)	4.82 (1.83)
		Slope (95% CI)	0.020 (−0.08, 0.121)	−0.107 (−0.486, 0.273)	0.117 (−0.242, 0.476)
	HIV−	Average, mean (SD)	6.82 (2.07)	4.79 (2.77)	6.19 (2.62)
		Slope (95% CI)	−0.030 (−0.263, 0.202)	0.052 (−0.858, 0.961)	0.064 (−0.796, 0.924)
		Slope Difference (95% CI)	0.023 (−0.152, 0.197)	−0.138 (−0.729, 0.453)	0.021(−0.616, 0.659)
Persistent heavy drinking	HIV+	Average, mean (SD)	11.56 (1.84)	7.31 (2.03)	11.03 (2.16)
		Slope (95% CI)	−0.043 (−0.157, 0.070)	0.670 (0.255, 1.086)	−0.185 (−0.637, 0.267)
	HIV−	Average, mean (SD)	16.58 (2.12)	12.43 (3.08)	19.75 (2.63)
		Slope (95% CI)	0.058 (−0.184, 0.300)	0.387 (−0.58, 1.354)	−0.577 (−1.476, 0.322)
		Slope Difference (95% CI)	−0.133 (−0.326, 0.060)	0.198 (−0.458, 0.855)	0.363 (−0.377, 1.103)

The column on the left represents the alcohol use group from the baseline period

*The average represents the estimated mean number of drinks per week at the start of the indicated time period. Standard errors are shown in parentheses. **The slope is an estimate of the change in drinks per week across the indicated time period. Confidence intervals are shown in parentheses. *** The slope difference represents the difference in the slopes between WLWH and WLWOH. A positive value indicates a faster increase among WLWH compared to WLWOH. Knots were introduced in the model at the start of each time period to allow for flexibility

**Table 4:** Linear mixed models examining demographic and clinical associations with alcohol consumption trends before and during the COVID-19 pandemic among women living with and without HIV

	Women living with HIV				Women living without HIV			
	Unadjusted		Adjusted		Unadjusted		Adjusted	
	Coefficient (CI 95%)	p-value	Coefficient (CI 95%)	p-value	Coefficient (CI 95%)	p-value	Coefficient (CI 95%)	p-value
Intercept	0.424 (-0.985, 1.833)	0.555	0.886 (-1.452, 3.224)	0.458	-0.142 (-3.919, 3.635)	0.941	1.409 (-3.995, 6.813)	0.609
Baseline Group								
Abstinence	REF		REF		REF		REF	
Non-heavy consumption	1.313 (-0.216, 2.842)	0.092	1.204 (-0.346, 2.754)	0.128	2.352 (-1.605, 6.309)	0.244	2.687 (-1.262, 6.636)	0.183
Some heavy consumption	6.265 (4.421, 8.109)	< 0.0001	5.892 (4.016, 7.768)	< 0.0001	6.030 (1.777, 10.283)	0.006	6.244 (1.989, 10.499)	0.004
Persistent heavy consumption	9.808 (7.597, 12.019)	< 0.0001	9.479 (7.249, 11.709)	< 0.0001	18.504 (13.959, 23.049)	< 0.0001	17.652 (13.119, 22.185)	< 0.0001
Pre-COVID-19 Time Segment	0.008 (-0.053, 0.069)	0.797	0.014 (-0.049, 0.077)	0.674	-0.020 (-0.175, 0.135)	0.801	-0.015 (-0.168, 0.138)	0.851
Pre-COVID-19 Time Segment*Group								
Pre-COVID-19*Abstinence	REF		REF		REF		REF	
Pre-COVID-19*Non-heavy consumption	0.014 (-0.053, 0.081)	0.686	0.012 (-0.057, 0.081)	0.739	0.045 (-0.118, 0.208)	0.585	0.048 (-0.113, 0.209)	0.559
Pre-COVID-19*Some heavy consumption	0.012 (-0.068, 0.092)	0.761	0.026 (-0.056, 0.108)	0.535	-0.011 (-0.185, 0.163)	0.906	-0.005 (-0.177, 0.167)	0.957
Pre-COVID-19*Persistent heavy consumption	-0.051 (-0.147, 0.045)	0.295	-0.050 (-0.148, 0.048)	0.321	0.078 (-0.108, 0.264)	0.412	0.038 (-0.146, 0.222)	0.682
Pandemic Onset Knot	0.317 (-2.072, 2.706)	0.795	0.273 (-2.185, 2.731)	0.828	-1.083 (-7.341, 5.175)	0.735	-1.162 (-7.330, 5.006)	0.712
Pandemic Onset Knot*Group								
Pandemic Onset Knot*Abstinence	REF		REF		REF		REF	
Pandemic Onset Knot*Non-heavy consumption	-0.408 (-3.017, 2.201)	0.759	-0.392 (-3.071, 2.287)	0.775	0.749 (-5.809, 7.307)	0.823	0.722 (-5.748, 7.192)	0.827
Pandemic Onset Knot*Some heavy consumption	0.260 (-2.933, 3.453)	0.873	0.057 (-3.224, 3.338)	0.973	-0.154 (-7.226, 6.918)	0.966	-0.241 (-7.254, 6.772)	0.946
Pandemic Onset Knot*Persistent heavy consumption	-5.426 (-9.207, -1.645)	0.005	-5.192 (-9.043, -1.341)	0.008	-5.431 (-13.136, 2.274)	0.167	-4.391 (-12.015, 3.233)	0.259
Early COVID-19 Time Segment	0.031 (-0.194, 0.256)	0.785	0.036 (-0.197, 0.269)	0.764	0.187 (-0.417, 0.791)	0.543	0.190 (-0.404, 0.784)	0.532
Early COVID-19 Time Segment*Group								
Early COVID-19*Abstinence	REF		REF		REF		REF	
Early COVID-19*Non-heavy consumption	-0.019 (-0.266, 0.228)	0.881	-0.022 (-0.277, 0.233)	0.867	-0.137 (-0.768, 0.494)	0.671	-0.138 (-0.761, 0.485)	0.664
Early COVID-19*Some heavy consumption	-0.138 (-0.444, 0.168)	0.375	-0.138 (-0.452, 0.176)	0.390	-0.136 (-0.816, 0.544)	0.696	-0.129 (-0.805, 0.547)	0.708
Early COVID-19*Persistent heavy consumption	0.639 (0.290, 0.988)	< 0.0001	0.607 (0.250, 0.964)	0.001	0.199 (-0.556, 0.954)	0.605	0.094 (-0.653, 0.841)	0.806
Mid-Pandemic Knot	-0.253 (-2.830, 2.324)	0.847	-0.275 (-2.896, 2.346)	0.837	-0.440 (-7.335, 6.455)	0.901	-0.612 (-7.403, 6.179)	0.860
Mid-Pandemic Knot*Group								
Mid-Pandemic Knot*Abstinence	REF		REF		REF		REF	
Mid-Pandemic Knot*Non-heavy consumption	0.399 (-2.406, 3.204)	0.781	0.342 (-2.514, 3.198)	0.815	1.295 (-5.918, 8.508)	0.725	1.641 (-5.474, 8.756)	0.651
Mid-Pandemic Knot*Some heavy consumption	-0.618 (-4.048, 2.812)	0.724	-0.595 (-4.086, 2.896)	0.738	1.200 (-6.618, 9.018)	0.764	1.155 (-6.579, 8.889)	0.770
Mid-Pandemic Knot*Persistent heavy consumption	-3.729 (-8.212, 0.754)	0.103	-3.635 (-8.172, 0.902)	0.116	2.747 (-5.767, 11.261)	0.527	4.237 (-4.160, 12.634)	0.323
Late COVID-19 Time Segment	0.029 (-0.192, 0.250)	0.797	0.027 (-0.198, 0.252)	0.817	-0.005 (-0.575, 0.565)	0.987	0.012 (-0.551, 0.575)	0.966
Late COVID-19 Time Segment*Group								
Late COVID-19*Abstinence	REF		REF		REF		REF	
Late COVID-19*Non-heavy consumption	-0.059 (-0.298, 0.180)	0.627	-0.049 (-0.290, 0.192)	0.691	-0.120 (-0.714, 0.474)	0.692	-0.155 (-0.741, 0.431)	0.604
Late COVID-19*Some heavy consumption	0.088 (-0.194, 0.370)	0.543	0.086 (-0.200, 0.372)	0.555	0.069 (-0.574, 0.712)	0.834	0.079 (-0.556, 0.714)	0.809
Late COVID-19*Persistent heavy consumption	-0.214 (-0.608, 0.180)	0.287	-0.194 (-0.592, 0.204)	0.340	-0.572 (-1.266, 0.122)	0.106	-0.567 (-1.251, 0.117)	0.104
Age, per 10 years			-0.191 (-0.503, 0.121)	0.230			-0.388 (-1.052, 0.276)	0.253
Marital Status								
Single/Divorced/Widowed			REF				REF	
Married/Partnered			-0.139 (-0.611, 0.333)	0.564			-0.278 (-1.140, 0.584)	0.528
Race								
White			REF				REF	
Black/African American			0.397 (-0.438, 1.232)	0.351			0.101 (-1.947, 2.149)	0.923
Other			0.924 (-0.005, 1.853)	0.052			0.015 (-2.139, 2.169)	0.989
Hispanic Ethnicity			-0.841 (-1.699, 0.017)	0.055			-0.031 (-1.726, 1.664)	0.972
Educational Attainment								
No high school diploma			REF				REF	
High school diploma			0.007 (-0.642, 0.656)	0.983			-1.077 (-2.506, 0.352)	0.140
Some college or more			-0.453 (-1.094, 0.188)	0.166			-0.809 (-2.214, 0.596)	0.260
Employed			0.100 (-0.357, 0.557)	0.668			-0.342 (-1.206, 0.522)	0.438
Annual Household Income								
<$12,000			REF				REF	
$12,000–30,000			-0.054 (-0.442, 0.334)	0.784			0.003 (-0.710, 0.716)	0.994
>$30,001			-0.221 (-0.731, 0.289)	0.395			0.407 (-0.534, 1.348)	0.397
Medical Comorbidity Burden (per 1 condition increase)			-0.026 (-0.171, 0.119)	0.723			0.250 (-0.097, 0.597)	0.157
Tobacco Smoking								
Never			REF				REF	
Former			0.691 (0.095, 1.287)	0.023			-1.018 (-2.466, 0.430)	0.169
Current			1.359 (0.761, 1.957)	< 0.0001			1.290 (-0.145, 2.725)	0.079

After adjusting for covariates, there was little change in the LMM (Table 4). In WLWH, current tobacco use was associated with overall increases in the average number of drinks per week during the study time period from 2017 to 2022 compared to women with no history of tobacco use (1.36 average drinks per week, 95% CI 0.761, 1.957). In WLWOH, there was no association with tobacco use and the average number of drinks per week (1.29 average drinks per week, 95% CI -0.145, 2.725) compared to women with no history of tobacco use.

## Discussion

Alcohol consumption remains a growing public health concern, especially among women, yet data on recent trends for WLWH and WLWOH in the U.S. are lacking. This study fills this gap by examining trends in alcohol use that span the pre-, early and late COVID-19 pandemic periods among a multi-site prospective cohort study of WLWH and socio-demographically similar WLWOH. Using longitudinal data from the WIHS, we captured longer-term alcohol use patterns and alcohol use history to identify drinking patterns that would otherwise be underappreciated using shorter time frames and more aggregate analytic approaches. We found that the amount of alcohol consumption varied by HIV status and previous alcohol use history, which could reflect differing drivers of alcohol use. Despite these differences, trends in alcohol use changes were similar between WLWH and WLWOH. This suggests that the pandemic did not have a unique impact on alcohol use among WLWH, but rather socio-economically disadvantaged women more broadly given that the trends observed in this cohort differed from previously observed national trends.

Unlike prior studies, we did not find overall higher alcohol consumption among WLWH compared to WLWOH [5]. Possible reasons include that WLWOH were enrolled based upon having similar risk behaviors and sociodemographic characteristics as WLWH and therefore do not represent the general population of US women. Furthermore, WIHS participants living with a long-standing HIV diagnosis may have higher levels of primary care engagement and access to services such as alcohol use disorder treatment [28]. Due to these features, WLWH may have had more counseling and treatment resources for unhealthy alcohol use, and greater awareness about health consequences of alcohol consumption especially in the setting of HIV infection, leading to behavior modification. There are also high levels of resilience among people living with HIV, which in turn may protect against maladaptive behaviors, such as unhealthy alcohol consumption [29–31]. Further research should explore protective factors to reduce unhealthy alcohol consumption in WLWH and socially vulnerable WLWOH.

Despite more self-reported alcohol consumption among WLWOH compared to WLWH, trends in alcohol use over the analysis period between the groups were similar. The largest changes in alcohol use following the COVID-19 pandemic occurred among those with a history of persistent heavy alcohol use, with initial decreases in use at the onset of the pandemic that substantially increased into 2021. A prior MWCCS analysis also found a reduction in binge drinking among PLWH at the onset of the pandemic [19]. Interestingly, the drinking declined during the late pandemic period in WLWH and WLWOH, suggesting that the increase in the early pandemic period was situational. These dynamic changes differ from nationally representative studies of U.S. adults where there were increases in alcohol use among women after the pandemic start and stability in alcohol consumption levels among women across the first year of the pandemic [16–18, 32]. Reasons for differences between WIHS participants and the general population are likely multifactorial and may reflect disproportionally high levels of economic strain, social isolation, and health concerns among WIHS participants as possible explanatory factors for the initial early pandemic decrease in alcohol use. Secondary increases in stress and anxiety are possible drivers for the subsequent increase in alcohol use [33].

Literature on alcohol use following economic crises and natural disasters has proposed household budget constraints as a mechanism for reduced alcohol use during periods of crisis [34, 35]. About half of WIHS participants are living below the poverty line and are therefore more susceptible to economic hardship. Participants with heavy drinking have a higher burden of alcohol expenses, which could explain why there was a large decrease in alcohol use following the onset of the pandemic among those with heavy drinking only. In a cross-sectional survey of U.S. adults following the pandemic onset (May 2020), nearly 13% reported decreased alcohol use citing financial concerns as one reason [36].

Social isolation and decreased access to bars early in the pandemic may be another contributing factor given that nearly all participants reported COVID-19 prevention behaviors [33]. As a result, there may have been decreases in heavy drinking due to limited access to alcohol and decreased opportunities for social drinking. Furthermore, participants have a high medical comorbidity burden and WLWH with heavy alcohol use have lower self-reported health status compared to those with non-heavy alcohol use [6]. Their perception of their health status may have increased personal concern for severe COVID-19 illness and therefore led to modifications in alcohol consumption and social behaviors during the early stages of the pandemic especially when there was a lack of comprehensive knowledge about the virus and no vaccines. This is supported by a qualitative study of PLWH in New York that found participants perceived themselves to be at greater risk for COVID-19 illness and therefore had high adherence to infection precautions [37].

Alcohol use trends have important implications for the public health community. For example, the initial decrease in alcohol use among those with histories of heavy drinking raise concern for alcohol withdrawal syndromes. In some communities there was an increase in hospitalizations associated with alcohol withdrawal during 2020, but not with alcohol use complications [38, 39]. These findings are consistent with reductions in consumption among those who had a history of heavy alcohol use and point to the need for public messaging on how to access formal treatment services and options for safely reducing consumption during periods of social disruption.

As the COVID-19 pandemic progressed, social restrictions lifted, COVID-19 vaccines were rolled out, and the above-mentioned drivers of the initial decrease in alcohol consumption may have been mitigated. However, during this time there was also an ongoing increase in stress and anxiety due to sequelae of the pandemic which may have driven the subsequent increase in alcohol use into 2021 [30, 32, 40–42]. During the late pandemic period, alcohol use declined among those with heavy drinking to below pre-pandemic levels. Longer-term trends will be needed to understand post-pandemic drinking patterns and if these changes are a function of decreased stressors, awareness of the health benefits of decreasing alcohol use, or increasing availability of treatment support following the relaxation of social restrictions and expansion of telehealth [43].

Other trends of interest include pandemic changes among those with a history of some heavy alcohol use where there was an increase in the average drinks per week throughout the late pandemic period in WLWH and WLWOH. Women in this alcohol use group may be progressing to unhealthy levels of alcohol use and need more support identifying their use as unhealthy and cutting back.

There was also a small increase in alcohol use after the pandemic start among those who previously were abstinent from alcohol and this increase persisted into 2022. Similar findings were observed among Mayo Clinic health network patients [44]. While the mean amount of alcohol in this group is well below national drinking guidelines for unhealthy use, initiation of drinking may still be cause for concern especially among this medically complex group. A large portion of alcohol-related harms occur among people who consume alcohol quantities below national low-risk drinking guidelines raising concern that smaller increases in drinking could increase medical complications [45]. Proactive efforts to prevent women from starting to consume alcohol are needed in addition to treating people who already consume unhealthy amounts of alcohol.

## Limitations

Alcohol use was self-reported and therefore subject to recall and social desirability bias, both of which could lead to an underestimation of alcohol use [46]. Non-response bias is also possible, but we did not find a correlation between the amount of missing alcohol data and number of drinks per week at other time points. There could be differences in reporting of alcohol use between the phone survey used in the pandemic and the in-person survey method used at the regular study visits, which could impact the trend observed at the pandemic onset. However, a prior study found no difference in self-reporting of alcohol use and related harms by phone versus in-person and previous estimates of WIHS alcohol consumption during the pandemic were found to be unbiased after adjusting for baseline demographic and clinical differences between those who did and did not respond to the survey early in the COVID-19 pandemic [47, 48]. There was a limited number of timepoints following the COVID-19 pandemic, which limits the generalizability of the study. Associations with stress, childcare responsibilities, anxiety and depression were not examined, which may provide further context on the trends observed.

## Conclusions

WLWH in WIHS from 2017 to 2022 consumed less alcohol on average compared to socio-demographically similar peers living without HIV, but alcohol use trajectories after the COVID-19 pandemic onset were similar between the two groups. Self-perceptions of health vulnerability and resilience from the experience of living with HIV are possible explanatory factors, but further research is needed to understand drivers for differences in quantities of alcohol consumption among WLWH and WLWOH. We also observed that trends varied by alcohol use history, highlighting the importance of examining alcohol use trends in the context of alcohol use history to avoid masking changes that may not be appreciated with more aggregated data analysis. Unique trends based on alcohol use histories should be used to inform targeted screening and messaging about alcohol use behaviors during future periods of social disruption and crisis. Longer term research is needed to examine if the COVID-19 pandemic has a lasting impact on the alcohol use trajectories of WLWH.

## Supplementary Information

Below is the link to the electronic supplementary material.

Supplementary Material 1

## Data Availability

Access to individual-level data from the MWCCS may be obtained upon review and approval of a MWCCS concept sheet. Links and instructions for online concept sheet submission are on the study website (http://mwccs.org/).
