# Supplementary material for: Trends in Alcohol Use Before and During the COVID-19 Pandemic Among Women Living With and Without HIV in the United States (2017–2022)
Source: AIDS Behav. 2025 Oct 10;30(2):502–15. doi: 10.1007/s10461-025-04875-9 (PMC12795451; doi:10.1007/s10461-025-04875-9)
Supplement: Supplementary file 1 — Supplementary Material 1 [file 10461_2025_4875_MOESM1_ESM.docx]

**Supplemental Tables and Figures**

**Supplemental Table 1. WIHS study visits included in the analysis**

| Study Time Period | WIHS Visit Number | Dates | Analysis Phase |
| --- | --- | --- | --- |
| Baseline | 41 | 10/01/2014-03/31/2015 | Baseline Alcohol Use Groups |
|  | 42 | 04/01/2015-09/30/2015 |  |
|  | 43 | 10/01/2015-03/31/2016 |  |
|  | 44 | 04/01/2016-09/30/2016 |  |
|  | 45 | 10/01/2016-03/31/2017 |  |
|  | 46 | 04/01/2017-09/30/2017 |  |
| Follow-up Period (Data included in the linear mixed model) | 47 | 10/01/2017-03/31/2018 | Pre-COVID-19 Pandemic |
|  | 48 | 04/01/2018-09/30/2018 |  |
|  | 49 | 10/01/2018-03/31/2019 |  |
|  | 50 | 04/01/2019-09/30/2019 |  |
|  | 100* | 03/01/2020-09/30/2020 | Early COVID-19 Pandemic |
|  | 101** | 10/01/2020-05/31/2021 |  |
|  | 101** | 06/01/2021-09/30/2021 | Late COVID-19 Pandemic |
|  | 102 | 10/01/2021-09/30/2022 |  |

* Data from this time point were collected via phone survey methods due to the COVID-19 public health emergency, while all other data were collected as part of an annual or semi-annual in-person study visit.

** Visit 101 occurred from 10/01/2020 to 09/30/21. Therefore, data from this visit contributed to both the early and the late COVID-19 pandemic time segments depending on when the participant’s visit occurred.

**Supplemental Table 2. Definitions for medical comorbidities**

| Comorbidity | Definition | Source | Comments |
| --- | --- | --- | --- |
| Cancer, non-AIDS | Breast, lung, colon, uterine, cervical, ovarian, liver, Hodgkin’s lymphoma | Self-report | Excluded skin cancer, Kaposi’s sarcoma, Non-Hodgkin’s lymphoma, central nervous system lymphoma, “metastatic cancer,” “other cancers” |
| Cardiovascular disease | Myocardial infarction or heart attack, revascularization or angioplasty, transient ischemic attack/stroke, angina or hospitalization for heart condition | Self-report | Included if any one of listed conditions or events was reported |
| Chronic kidney disease | eGFR <60 mL/min/1.73 m^2^  (determined by the CKD-epi formula) | Laboratory | Abnormal on two consecutive study visits |
| Diabetes mellitus, type 2 | Receipt of anti-diabetic medication  *Or* FBG ≥126 mg/dL on two study visits  *Or* HgbA1c ≥6.5% and FBG above threshold at one study visit | Self-report or laboratory | N/A |
| Dyslipidemia | Receipt of lipid-lowering medication  *Or* LDL ≥130 mg/dL  *And* HDL <40 mg/dL | Self-report or laboratory | N/A |
| Hypertension | Receipt of anti-hypertensive medication  *Or* elevated blood pressure on any two study visits  (systolic ≥140 and/or diastolic ≥90) | Self-report or clinical measurement | N/A |
| Liver disease | Chronic hepatitis B or C viral infection  *Or* FIB-4 score >3.25  *Or* Fibroscan score ≥ 12.5 | Laboratory or clinical measurement | N/A |
| Lung disease | Asthma, chronic obstructive pulmonary disease, or hospitalization for a lung problem other than pneumonia | Self-report | Included if any one of listed diagnoses was reported |

Adapted from Collins LF, Sheth AN, Mehta CC, et al. The Prevalence and Burden of Non-AIDS Comorbidities Among Women Living With or at Risk for Human Immunodeficiency Virus Infection in the United States. Clin Infect Dis. 2021;72(8):1301-1311


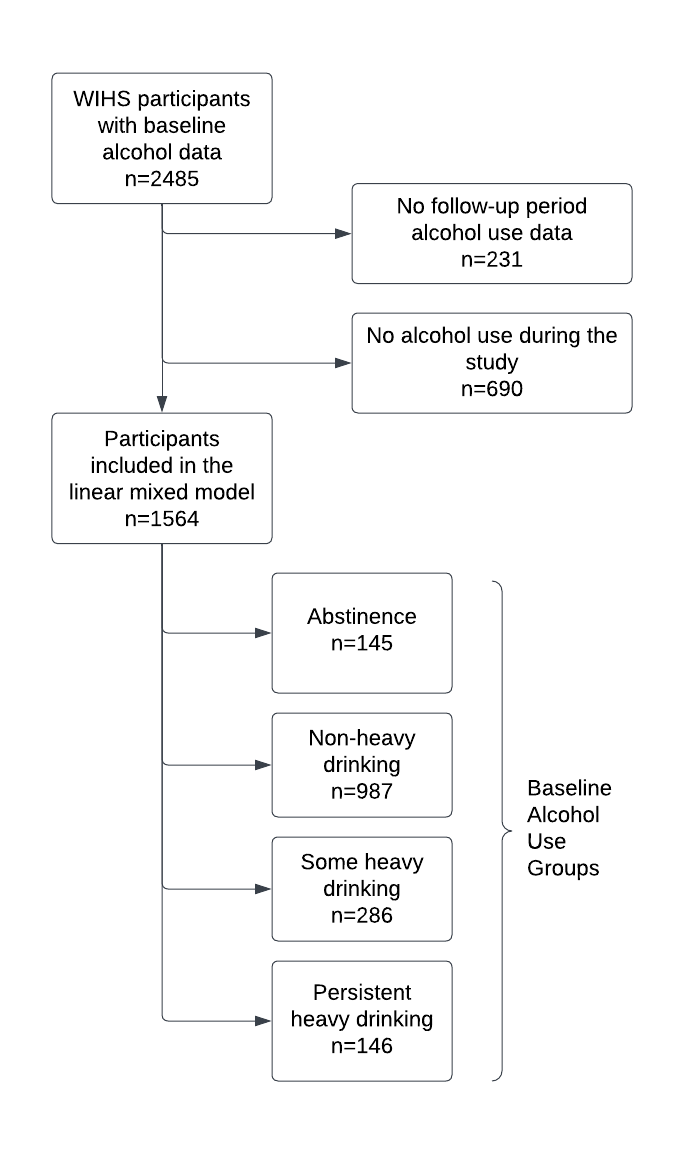


**Supplemental Fig 1.** Participant Flow Diagram for women living with and out HIV in the Women’s Interagency Study

**Supplemental Table 3. Demographics and clinical characteristics by baseline alcohol consumption group for women living with and without HIV in the Women’s Interagency Study**

| Measure | Total | Baseline Groups | | | | Excluded | P-value |
| --- | --- | --- | --- | --- | --- | --- | --- |
|  |  | **Abstinence** | **Non-heavy Consumption** | **Some Heavy Consumption** | **Persistent Heavy Consumption** |  |  |
| Total number | 2254 | 145 | 987 | 286 | 146 | 690 |  |
| N (%) or mean (sd) |  |  |  |  |  |  |  |
| HIV Positive | 1582 (70.2%) | 117 (80.7%) | 681 (69.0%) | 167 (58.4%) | 82 (56.2%) | 535 (77.5%) | <0.0001² |
| Age, years | 50.4 ± 9.38 | 51.6 ± 8.53 | 49.4 ± 9.57 | 48.3 ± 9.20 | 50.0 ± 8.29 | 52.6 ± 9.14 | <0.0001¹ |
| Study Site |  |  |  |  |  |  |  |
| Bronx | 316 (14.0%) | 22 (15.2%) | 124 (12.6%) | 43 (15.0%) | 16 (11.0%) | 111 (16.2%) | <0.0001² |
| Brooklyn | 314 (14.0%) | 29 (20.0%) | 156 (15.8%) | 27 (9.4%) | 13 (8.9%) | 89 (13.0%) |  |
| Washington DC | 278 (12.4%) | 15 (10.3%) | 125 (12.7%) | 31 (10.8%) | 19 (13.0%) | 88 (12.8%) |  |
| San Francisco | 303 (13.5%) | 8 (5.5%) | 136 (13.8%) | 39 (13.6%) | 23 (15.8%) | 97 (14.1%) |  |
| Chicago Cook County | 261 (11.6%) | 27 (18.6%) | 112 (11.3%) | 29 (10.1%) | 11 (7.5%) | 82 (12.0%) |  |
| Chapel Hill | 185 (8.2%) | 7 (4.8%) | 78 (7.9%) | 27 (9.4%) | 17 (11.6%) | 56 (8.2%) |  |
| Atlanta | 260 (11.6%) | 8 (5.5%) | 119 (12.1%) | 42 (14.7%) | 27 (18.5%) | 64 (9.3%) |  |
| Miami | 127 (5.6%) | 11 (7.6%) | 37 (3.7%) | 17 (5.9%) | 10 (6.8%) | 52 (7.6%) |  |
| Birmingham | 102 (4.5%) | 11 (7.6%) | 50 (5.1%) | 11 (3.8%) | 4 (2.7%) | 26 (3.8%) |  |
| Jackson | 104 (4.6%) | 7 (4.8%) | 50 (5.1%) | 20 (7.0%) | 6 (4.1%) | 21 (3.1%) |  |
| Marital Status |  |  |  |  |  |  |  |
| Married/Partnered | 649 (30.4%) | 36 (26.1%) | 290 (30.7%) | 80 (29.3%) | 46 (31.9%) | 197 (30.9%) | 0.80² |
| Single/Divorced/Widowed | 1489 (69.6%) | 102 (73.9%) | 655 (69.3%) | 193 (70.7%) | 98 (68.1%) | 441 (69.1%) |  |
| *Missing* | *116* | *7* | *42* | *2* | *13* | *52* |  |
| Race |  |  |  |  |  |  |  |
| Black/African American | 1404 (62.3%) | 96 (66.2%) | 615 (62.3%) | 190 (66.4%) | 100 (68.5%) | 403 (58.4%) | 0.23² |
| White | 245 (10.9%) | 14 (9.7%) | 106 (10.7%) | 27 (9.4%) | 16 (11.0%) | 82 (11.9%) |  |
| Other | 605 (26.8%) | 35 (24.1%) | 266 (27.0%) | 69 (24.1%) | 30 (20.5%) | 205 (29.7%) |  |
| Hispanic Ethnicity | 337 (15.0%) | 22 (15.2%) | 133 (13.5%) | 35 (12.2%) | 17 (11.6%) | 130 (18.8%) | 0.012² |
| Employed | 890 (39.9%) | 41 (28.3%) | 446 (45.5%) | 120 (42.0%) | 62 (42.5%) | 221 (32.9%) | <0.0001² |
| *Missing* | *25* | *0* | *7* | *0* | *0* | *18* |  |
| Annual Household Income |  |  |  |  |  |  |  |
| <$12000 | 976 (48.1%) | 84 (60.4%) | 378 (41.9%) | 139 (52.5%) | 64 (46.4%) | 311 (53.1%) | <0.0001² |
| $12000-30000 | 587 (28.9%) | 36 (25.9%) | 280 (31.0%) | 61 (23.0%) | 46 (33.3%) | 587 (28.9%) |  |
| >$30001 | 468 (23.0%) | 19 (13.7%) | 245 (27.1%) | 65 (24.5%) | 28 (20.3%) | 111 (18.9%) |  |
| *Missing* | *223* | *6* | *84* | *21* | *8* | *104* |  |
| Educational Attainment |  |  |  |  |  |  |  |
| No high school diploma | 686 (32.7%) | 51 (36.2%) | 264 (28.4%) | 99 (36.1%) | 56 (39.2%) | 216 (35.4%) | <0.0001² |
| High school diploma | 659 (31.4%) | 46 (32.6%) | 280 (30.1%) | 85 (31.0%) | 34 (23.8%) | 214 (35.1%) |  |
| Some college or more | 754 (35.9%) | 44 (31.2%) | 387 (41.6%) | 90 (32.8%) | 53 (37.1%) | 180 (29.5%) |  |
| *Missing* | *155* | *4* | *56* | *12* | *3* | *80* |  |
| Chronic medical comorbidities | 3.35 ± 2.00 | 3.86 ± 1.99 | 3.13 ± 2.01 | 3.12 ± 1.82 | 3.18 ± 1.73 | 3.68 ± 2.04 | <0.0001¹ |
| Visits completed during the follow-up/COVID-19 time period | 5.44 ± 1.81 | 6.25 ± 1.06 | 5.73 ± 1.56 | 5.57 ± 1.61 | 5.84 ± 1.38 | 4.72 ± 2.17 | <0.0001¹ |

^1^The Kruskal-Wallis test was used to compare means across alcohol consumption groups

^2^Chi-squared tests were used to compare proportions across alcohol consumption groups.

The “Excluded” column represents participants who did not report any drinking across the entire follow-up period that included the COVID-19 pandemic, and were therefore excluded from the LMM. The other alcohol consumption groups are based on a participant’s consumption pattern during the baseline period; these participants were included in the LMM of the COVID-19 pandemic time period. If data were missing, values were carried forward from a recent visit. Remaining missing data is listed in the table.

**Supplemental Table 4. Linear mixed model estimates of the number of drinks per week before and during the COVID-19 Pandemic among women living with and without HIV with HIV interaction term**

|  | Coefficient (95% CI) | P-value |
| --- | --- | --- |
| Intercept | -0.143 (-3.334, 3.048) | 0.930 |
| HIV Status | 0.567 (-2.989, 4.122) | 0.755 |
| Baseline Group |  |  |
| Abstinence | REF |  |
| Non-heavy consumption | 2.337 (-1.008, 5.682) | 0.171 |
| Some heavy consumption | 6.042 (2.446, 9.638) | 0.001 |
| Persistent heavy consumption | 18.520 (14.679, 22.362) | <.0001 |
| Baseline Group*HIV |  |  |
| Abstinence*HIV | REF |  |
| Non-heavy consumption*HIV | -1.017 (-4.769, 2.735) | 0.595 |
| Some heavy consumption*HIV | 0.254 (-3.886, 4.394) | 0.904 |
| Persistent heavy consumption*HIV | -8.746 (-13.307, -4.185) | <0.001 |
| Pre-COVID-19 Time Segment | -0.020 (-0.154, 0.114) | 0.771 |
| Pre-COVID-19 Time Segment*HIV | 0.028 (-0.122, 0.177) | 0.715 |
| Pre-COVID-19 Time Segment*Group |  |  |
| Pre-COVID-19*Abstinence | REF |  |
| Pre-COVID-19*Non-heavy consumption | 0.045 (-0.096, 0.186) | 0.533 |
| Pre-COVID-19*Some heavy consumption | -0.009 (-0.161, 0.142) | 0.904 |
| Pre-COVID-19*Persistent heavy consumption | 0.080 (-0.082, 0.242) | 0.335 |
| Pre-COVID-19 Time Segment*Group*HIV |  |  |
| Pre-COVID-19*Abstinence*HIV | REF |  |
| Pre-COVID-19*Non-heavy consumption*HIV | -0.031 (-0.189, 0.127) | 0.703 |
| Pre-COVID-19*Some heavy consumption*HIV | 0.023 (-0.152, 0.197) | 0.800 |
| Pre-COVID-19*Persistent heavy consumption*HIV | -0.133 (-0.326, 0.060) | 0.176 |
| Pandemic Onset Knot | -1.084 (-6.522, 4.355) | 0.696 |
| Pandemic Onset Knot*HIV* | 1.399 (-4.628, 7.426) | 0.649 |
| Pandemic Onset Knot*Group |  |  |
| Pandemic Onset Knot*Abstinence | REF |  |
| Pandemic Onset Knot*Non-heavy consumption | 0.739 (-4.959, 6.436) | 0.799 |
| Pandemic Onset Knot*Some heavy consumption | -0.173 (-6.317, 5.971) | 0.956 |
| Pandemic Onset Knot*Persistent heavy consumption | -5.496 (-12.191, 1.199) | 0.108 |
| Pandemic Onset Knot*Group*HIV |  |  |
| Pandemic Onset Knot*Abstinence*HIV | REF |  |
| Pandemic Onset Knot*Non-heavy consumption*HIV | -1.153 (-7.518, 5.212) | 0.723 |
| Pandemic Onset Knot*Some heavy consumption*HIV | 0.439 (-6.618, 7.495) | 0.903 |
| Pandemic Onset Knot*Persistent heavy consumption*HIV | 0.089 (-7.768, 7.945) | 0.982 |
| Early COVID-19 Time Segment | 0.188 (-0.337, 0.712) | 0.483 |
| Early COVID-19 Time Segment*HIV | -0.156 (-0.735, 0.423) | 0.597 |
| Early COVID-19 Time Segment*Group |  |  |
| Early COVID-19*Abstinence | REF |  |
| Early COVID-19*Non-heavy consumption | -0.135 (-0.684, 0.414) | 0.629 |
| Early COVID-19*Some heavy consumption | -0.138 (-0.729, 0.453) | 0.647 |
| Early COVID-19*Persistent heavy consumption | 0.198 (-0.458, 0.855) | 0.554 |
| Early COVID-19 Time Segment*Group*HIV |  |  |
| Early COVID-19*Abstinence*HIV | REF |  |
| Early COVID-19*Non-heavy consumption*HIV | 0.117 (-0.495, 0.728) | 0.708 |
| Early COVID-19*Some heavy consumption*HIV | -0.002 (-0.680, 0.676) | 0.996 |
| Early COVID-19*Persistent heavy consumption*HIV | 0.445 (-0.313, 1.202) | 0.250 |
| Mid-Pandemic Knot | -0.446 (-6.430, 5.538) | 0.884 |
| Mid-Pandemic Knot*HIV | 0.194 (-6.416, 6.803) | 0.954 |
| Mid-Pandemic Knot*Group |  |  |
| Mid-Pandemic Knot*Abstinence | REF |  |
| Mid-Pandemic Knot*Non-heavy consumption | 1.278 (-4.981, 7.537) | 0.689 |
| Mid-Pandemic Knot*Some heavy consumption | 1.238 (-5.548, 8.023) | 0.721 |
| Mid-Pandemic Knot*Persistent heavy consumption | 2.809 (-4.581, 10.198) | 0.456 |
| Mid-Pandemic Knot*Group*HIV |  |  |
| Mid-Pandemic Knot*Abstinence*HIV | REF |  |
| Mid-Pandemic Knot*Non-heavy consumption*HIV | -0.891 (-7.854, 6.073) | 0.802 |
| Mid-Pandemic Knot*Some heavy consumption*HIV | -1.853 (-9.597, 5.892) | 0.639 |
| Mid-Pandemic Knot*Persistent heavy consumption*HIV | -6.546 (-15.400, 2.307) | 0.147 |
| Late COVID-19 Pandemic Time Segment | -0.005 (-0.500, 0.491) | 0.986 |
| Late COVID-19 Pandemic Time Segment*HIV | 0.034 (-0.517, 0.585) | 0.904 |
| Late COVID-19 Pandemic Time Segment*Group |  |  |
| Late COVID-19*Abstinence | REF |  |
| Late COVID-19*Non-heavy consumption | -0.120 (-0.636, 0.396) | 0.649 |
| Late COVID-19*Some heavy consumption | 0.067 (-0.492, 0.625) | 0.815 |
| Late COVID-19*Persistent heavy consumption | -0.575 (-1.177, 0.028) | 0.062 |
| Late COVID-19 Pandemic Time Segment*Group*HIV |  |  |
| Late COVID-19*Abstinence*HIV | REF |  |
| Late COVID-19*Non-heavy consumption*HIV | 0.062 (-0.516, 0.639) | 0.834 |
| Late COVID-19*Some heavy consumption*HIV | 0.021 (-0.616, 0.659) | 0.948 |
| Late COVID-19*Persistent heavy consumption*HIV | 0.363 (-0.377, 1.103) | 0.336 |

END
